# Systematic Review of Outcome Measures Reported in Clinical Canine Osteoarthritis Research

**DOI:** 10.1111/vsu.12479

**Published:** 2016-04-27

**Authors:** Zoe Belshaw, Lucy Asher, Rachel S. Dean

**Affiliations:** ^1^ Centre for Evidence‐based Veterinary Medicine, School of Veterinary Medicine and Science, University of NottinghamUnited Kingdom; ^2^ School of Veterinary Medicine and ScienceUniversity of NottinghamUnited Kingdom

## Abstract

**Objective:**

To record and categorize the outcome measures used in dogs with naturally occurring osteoarthritis (OA) by systematically reviewing the peer reviewed publications on OA in dogs.

**Study Design:**

Systematic literature review.

**Study Population:**

Peer reviewed literature on canine OA.

**Methods:**

A computer‐based bibliographic search was performed on PubMed and CAB Abstracts in August 2013 to find peer reviewed publications relevant to canine OA. Inclusion and exclusion criteria were applied. The outcome measures reported within each publication were recorded and categorized for comparison. Adequately described outcome measures were assessed for uniqueness and evidence of prior validation.

**Results:**

Of 3,697 publications identified and screened, 117 were deemed eligible for inclusion. Within eligible publications, outcome measures were used 618 times (median of 4 outcome measures per publication). Outcomes measured were divided into 5 groups containing 65 categories. The most frequently assessed outcomes were lameness assessment with no stated gait/mixed gaits (66 outcomes), radiography (58), and lameness single gait/lateral motion (55). Of 618 outcome measures reported, 491 were assessed for uniqueness and 348 (71%) were unique to a single publication. Ten outcome measures were reported to have been validated.

**Conclusion:**

Many outcome measures have been used to assess canine OA. There is no consensus on which are the most useful outcomes or by which method they should be assessed. There is a pressing need for agreement on outcomes reporting in canine OA and for validation of outcome measures used for these assessments. Until consensus is reached, we recommend at least one validated outcome measure be used in every clinical study.

The efficacy of clinical interventions in veterinary medicine and surgery are appraised by measuring outcomes, which are often diverse and may be specific to an intervention (eg, prevalence of a certain side effect) or may be broad measures applicable to multiple diseases and interventions (eg, quality of life or survival time). The terms outcome instrument^1^ (typically referring to a patient‐completed questionnaire) or outcome measure are used to describe a specific tool used to provide data that measure a specific outcome.^2^ In veterinary medicine, outcome measures are typically completed by an owner or veterinarian. Musculoskeletal disease is a common complaint in dogs presented to veterinarians^3^ and osteoarthritis (OA) is a common canine disease^2^ for which good outcome measures are lacking.^4^

Individual studies that examine the same research question can be identified, interpreted, compared, and summarized in a process called evidence or research synthesis. The most frequently used method of evidence synthesis is the systematic review.^5^, ^6^ To enable conclusions to be drawn in a systematic review, outcomes assessed and outcome measures used for these assessments in different studies should be sufficiently similar to allow comparison of the results generated. Unfortunately, the quality of the design and reporting of veterinary clinical trials is frequently too poor for definitive conclusions to be drawn when systematic reviews are conducted.^7^ Systematic reviews of the efficacy of OA treatments in dogs highlight the poor quality of study design and reporting, limiting the ability of their authors to make strong recommendations.^8^, ^9^, ^10^ An important factor in the quality of any outcome assessment is the validity of the outcome measure used for that assessment.^1^ Application of unvalidated outcome measures risks the collection and dissemination of inaccurate or irrelevant data.^1^, ^11^ Validated veterinary outcome measures exist, but are infrequently used.^4^, ^7^

There has not been a systematic review of the outcome measures used in veterinary medicine to assess any disease. A review of this nature on the subject of OA in people raised awareness of the multiplicity and poor validation of outcome measures,^12^ which led to a consensus on standardized outcome measures for use in all clinical studies of hip and knee OA^13^ and on reporting of histopathological changes in canine models of OA.^14^ The need for improvement of veterinary outcome reporting has been described by the Canine Orthopedic Outcome Measures Program (COMP).^1^, ^15^, ^16^, ^17^, ^18^ However, the outcomes most frequently measured, the methods of their assessment, and the frequency of reference to the need for their validation have not been reported for canine OA. There is therefore a need for a systematic review of the outcome measures used in this field.

The aim of this review is to describe the use and nature of outcome measures in the peer reviewed literature on canine OA. The objective was to record and categorize the outcome measures used in dogs with naturally‐occurring OA by systematically reviewing the peer reviewed publications on OA in dogs.

## Materials and Methods

### Search Strategy

Searches of PubMed (1948–2013) and CAB Abstracts (1910–2013) were performed in August 2013 using the OVID interface. The abstract, original title, broad terms, and key words were searched using terms relevant to dogs (dog, dogs, canine, canines, or *canis*) and OA (arthritis, osteoarthritis, OA, degenerative joint disease (DJD), degenerative joint, degenerative articular). The searches were linked with Boolean terms ([dog OR dogs OR canine OR canines OR *canis*] AND [arthritis OR OA OR DJD OR degenerative joint OR degenerative articular]).

### Inclusion and Exclusion Criteria

The inclusion criteria were that the publication must: (1) be in the English language; (2) be in a peer reviewed journal; (3) be accessible by the authors through institutional access, internet searching, or contacting the authors; (4) contain 1 of the search terms in the title, key words, or abstract; (5) be a primary research publication (ie, original scientific research); (6) describe dogs with naturally occurring OA; and (7) use at least 1 outcome measure for assessment of canine OA. The exclusion criteria were any publications that did not meet the inclusion criteria plus those that involved cases of infectious or immune‐mediated arthritis or where the main focus was not on OA. A single author (ZB) performed the initial search and applied inclusion and exclusion criteria to all publications. For consistency, a random sample of 20% of all publications that met the inclusion criteria was independently screened according to the exclusion criteria by a second author (RD).

### Evaluation Criteria

An outcome measure was defined a specific measurement used to provide data that assessed a specific outcome in clinical canine OA. A single question in a multi‐question outcome measure was defined as an item. The number of outcome measures used in each included publication was recorded as were details of the specific outcomes assessed and the methodologies used for these assessments.

Outcomes assessed were split into 5 groups inductively developed by the authors (Table 1). Within these groups, outcomes were categorized according to the assessment being made (eg, activity, crepitus, and lameness). All outcomes were placed into their categories by 1 author (ZB). Where the categorization of an outcome was difficult, its grouping was discussed with a second author (LA) and a consensus was reached. Outcome measures used for the assessment of each outcome were then collated. Where, for example, a lameness score was composed of the summed scores of 5 components, these components were counted as separate outcome measures, because in some publications the results of the individual components were discussed as separate results. Outcome measures in the “Named Measures” category were not split into their individual items and were therefore each counted as a single outcome measure. This was because these outcome measures were all validated to be used in their entirety and therefore should not be reported in terms of their individual component items. The gaits used in outcome measures categorized as “limb only physical examination” were recorded separately, as several publications assessed and reported multiple individual gaits or directions of travel. Orthopedic and neurological examinations were included only when specific measurements were described (eg, joint range of motion, muscle circumference).

**Table 1:** Summary of outcome measures reviewed.

Outcome group	Outcome	Pubs used to assess outcome	Unique outcome measures	Outcome measures incompletely described
Named measures (multi‐item questionnaires with a specific name)	Canine Brief Pain Inventory (CBPI)	10	1	0
	GUVQuest	3	1	0
	Helsinki Chronic Pain Index (HCPI)	6	1	0
	Liverpool Osteoarthritis in Dogs (LOAD) clinical metrology instrument	2	1	0
Behavioral/welfare (assessments of specific behaviors or welfare indicators)	Activity	16	8	6
	Aggression	5	3	0
	Attitude	4	2	2
	Change over time	33	21	5
	Client specific outcome measure (CSOM)	5	2	0
	Comfort	2	2	0
	Contact with owners	1	1	0
	Following owners	1	1	0
	Happiness	1	1	0
	Owner questionnaire (novel)	4	1	3
	Pain	54	35	4
	Play	10	9	0
	Quality of life	7	5	1
	Severity of disease	1	1	0
	Submissiveness	1	1	0
	Tail wag	1	1	0
	Vocalization	3	3	0
Limb only physical examination (clinical veterinary assessments of limb anatomy or function)	Contralateral limb lift	10	8	1
	Crepitus	6	6	0
	Goniometry	14	8	1
	Joint stability	3	2	1
	Limb circumference—forelimb	1	1	0
	Limb circumference—hindlimb	3	2	1
	Muscle atrophy	1	1	0
	Patellar luxation	1	1	0
	Proprioception	1	1	0
	Range of motion	23	18	1
	Swelling	9	8	0
	Weakness	1	1	0
	Weight bearing	19	13	0
	Withdrawal	1	1	0
Visually observed mobility (assessments of gait or mobility)	Car—get into/out of	3	3	0
	Climate (influence of on dog's mobility)	1	1	0
	Exercise tolerance	2	2	0
	Jump	14	11	3
	Lame (no stated gait/mixed gaits)	66	49	3
	Lame (single gait/lateral motion)	55	45	2
	‐ (Lame diagonal walk)	(1)	(1)	(0)
	‐ (Lame gallop)	(3)	(3)	(0)
	‐ (Lame run)	(11)	(8)	(0)
	‐ (Lame trot)	(14)	(12)	(1)
	‐ (Lame turn)	(2)	(1)	(0)
	‐ (Lame walk)	(24)	(20)	(1)
	Lie down	5	4	0
	Mobility impairment	6	6	0
	Pace on a walk	4	3	0
	Paralysis	3	1	2
	Rise from sit/lie	16	13	1
	Sit	1	1	0
	Stair/ramp ascend or descend	27	20	0
	Stand or lie	1	1	0
	Stiffness	22	15	2
Outcomes from advanced veterinary diagnostic investigations	Accelerometry	7	3	1
	Arthroscopy	1	*	*
	Arthrotomy	1	*	*
	Computer tomography	3	*	*
	Electrodermal testing	1	*	*
	Electromyelography	1	*	*
	Force plate gait analysis	42	*	*
	Hormonal tests	5	*	*
	Kinematics	3	*	*
	Neurological examination	1	*	*
	Orthopedic examination	1	*	*
	Radiography	58	*	*
	Scintigraphy	1	*	*
	Surface electromyelography	1	*	*
	Synovial fluid assessment	3	*	*
	**Total**	618	348/491 [127 not assessed]	40

*Refers to outcome measures not assessed for uniqueness due to their complex methodology.

The number of times each individual outcome measure was used across publications was also ascertained. When an outcome measure was used in only 1 publication, this was described as a “unique” outcome measure. Uniqueness was assessed by 1 author (ZB) manually comparing all reported outcome measures for assessment of each separate outcome. An example of uniqueness would be an outcome measure assessing lameness using a numeric scale of 1–7 that was used in only 1 publication. Outcome measures for the assessment of most of the “outcomes from advanced veterinary diagnostic investigations” group were not assessed for uniqueness since the methodologies used were complex and were reported to different levels of detail. As a basic assessment of the use of validated outcome measures, the publications were searched for use of the term “valid*” (where the * is an abbreviation allowing any words starting with “valid” to be found). Any outcome measures said to be validated by the authors were recorded; however, the quality of the validation process was not assessed. Where a reference to another publication was included with an outcome measure methodology, this was noted. Where available, that publication was read to determine whether it included any reference of the measure being validated.

## Results

**Figure 1:**
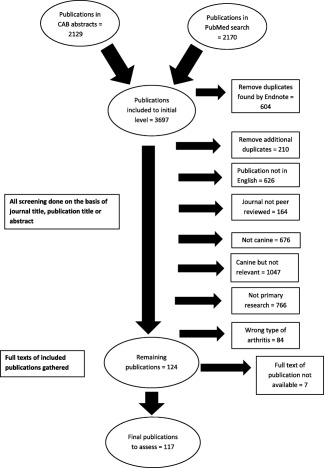
Summary of the systematic review process.

From a total of 3,697 publications found in the initial search 117 publications were deemed eligible for inclusion (Fig 1). In these 117 publications, outcome measures were used 618 times (Table 1). The number of outcomes assessed per study ranged from 1 to 32 with a mode of 1 and a median of 4 (IQR ± 4).

The 117 publications dated from 1954 to 2013 and were found in 25 journals. Sixteen publications either described the clinical manifestations of OA or assessed risk factors for its development: 67 publications reported the effect of treatment on dogs with OA, 4 described diagnostic methods, 18 evaluated or compared outcome measures, 2 assessed monitoring methods, 8 assessed the impact of OA on gait, and 2 assessed the impact of OA on radiological measurements.

Outcome measures were divided into 5 groups and further divided into 65 categories (excluding subsets of “limb only physical examination”; Table 1) according to the outcome they assessed. The most frequently assessed outcomes were “lame (no stated gait/mixed gaits)” (66 outcomes), “radiography” (58), “lame (single gait/lateral motion)” (55), and “pain” (54). The 618 outcome measures were used by veterinarians (356), owners (243), veterinary physical rehabilitation practitioners (14), veterinarian and owner together (2), or researchers (2). The user was unclear for 1 assessment.

Of the 618 outcome measures reported, 491 were suitable for assessment of uniqueness. The remaining 127 outcome measures were too complex for evaluation as described in the evaluation criteria. Of these 491 outcome measures, 348 (71%) were found to be unique to a single publication and a further 40 (8%) were not described in enough detail to be assessed for uniqueness. The majority of categories included multiple unique outcome measures. For example, “lame (no stated gait/mixed gaits)” (66 outcomes) was assessed using 49 different unique outcome measures. Response options included visual analogue scales (VAS), numeric scales with descriptors, descriptors only, and a combination of methods. Within the numeric scales with descriptors used for this outcome measure alone, 11 different numeric scoring systems were used (eg, 0–3, 1–5, 1–7) and several different descriptors were reported for the same numeric score. This phenomenon was similar across most categories. The emphasis of the outcome measure was also variable. As examples, “play” encompassed assessments of ability, willingness, improvement, change, or hesitation. “Change over time” included binary, numeric, VAS scales, and descriptor scales to determine owner and veterinarian satisfaction with improvement, relative improvement since the last assessment, and whether or not the dog had been “cured.”

Force plate gait analysis and radiography were the most commonly reported single outcomes in the 26/117 publications where only 1 outcome was assessed. Outcome measures used for the assessment of these outcomes were not assessed for uniqueness due to their variable levels of methodological description. Force plate gait analysis methodology was not standardized, was variably reported, and poorly referenced. In some studies,^19^, ^20^ force plate gait analysis was the only outcome assessed. Many publications combined force plate gait analysis as an objective gold standard with other outcomes (eg, lameness scores and owner questionnaires).^21^ Radiography was used either as an additional inclusion criterion after study enrollment or to subjectively or objectively check the progress of a particular type of pathology. Many different radiological outcome measures were used with little reference to their validity.^22^, ^23^, ^24^

Reference to the validation of an outcome measure was absent in almost all publications other than to state that validated outcome measures did not exist for the desired outcome.^4^ Seven publications^11^, ^31^, ^36^, ^37^, ^38^, ^39^, ^40^ reported 10 outcome measures as being validated (Table 2) and 5 of these outcome measures were for owner assessments. These validated outcome measures were seldom used other than by their authors. Serial publications from the same clinical study frequently accounted for the same outcome measure being used in more than 1 publication.^25^, ^26^, ^27^ Fifty of 117 publications included a reference associated with the outcome measure of 1 or more outcomes. Most of the references that were available had used the same outcome measure but did not provide any evidence of validation. Thirteen of 50 publications containing references stated that the authors had modified the outcome measure to which they referred, invalidating any prior validation.

**Table 2:** Outcome measures reported to be validated.

Outcome group	Outcome	Outcome measure	Reference
Named measures	Canine Brief Pain Inventory (CBPI)	Canine Brief Pain Inventory (CBPI)	Brown^31^
Named measures	Helsinki Chronic Pain Index (HCPI)	Helsinki Chronic Pain Index (HCPI)	Hielm‐Bjorkman^37^
Named measures	Liverpool Osteoarthritis in Dogs (LOAD) clinical metrology instrument	Liverpool Osteoarthritis in Dogs (LOAD) clinical metrology instrument	Walton^11^
Named measures	Glasgow University Veterinary School Questionnaire (GUVQuest)	Glasgow University Veterinary School Questionnaire (GUVQuest)	Wiseman‐Orr^40^
Behavioral/welfare assessment	Pain	Visual analogue scale questionnaire	Hudson^39^
Limb‐only physical examination	Sit	Asymmetry in standing and lying position	Hyytiainen^38^
Limb‐only physical examination	Muscle atrophy	Assessment of muscle atrophy	Hyytiainen^38^
Limb‐only physical examination	Weight bearing	Manual and measured static weight bearing	Hyytiainen^38^
Limb‐only physical examination	Range of motion	Measurement of stifle range of motion	Hyytiainen^38^
Outcomes from advanced veterinary diagnostic investigations	Accelerometry	Activity monitor	Brown^36^

## Discussion

This systematic review demonstrates that many outcomes are assessed in the study of clinical canine OA and that these assessments are made using multiple unique outcome measures. Few outcome measures were identified as being validated and these validated outcome measures were rarely used. The use of numerous unvalidated outcome measures may lead to unreliable, unrepeatable results that are too different to be compared. Recommendations based on these studies that may be subsequently widely adopted may be unsubstantiated and potentially unsafe.^28^

Our results illustrate that there is no consensus on which outcomes should be assessed in canine OA or which outcome measures should be used. Visual lameness assessment, radiographic appearance, and an assessment of pain were the three most commonly assessed outcomes found by our review. This is not surprising, as lameness and pain are likely to be of importance to owners and radiography is one of the few objective assessments available to veterinarians assessing these dogs in first‐opinion practice. A recent narrative review of some of the outcome measures used in canine OA highlighted that no single outcome truly captures the complexity of this disease.^29^ This complexity likely explains, at least in part, the multitude of different outcomes that are assessed and reinforces that more work is needed to determine which outcomes best reflect relevant clinical change.

The same review as that described above adds weight to the argument that validated outcome measures must be used and that validated outcome measures should be developed for all frequently assessed outcomes.^29^ Since the search for our review was conducted, outcome measures for the assessment of lameness^30^ and pain^31^ have been validated for completion by owners, but not veterinary surgeons. In human orthopedic studies, patient‐reported outcomes have been recommended as the gold standard.^32^ The validation of outcome measures for completion by owners is welcomed; however, some veterinarians may feel uncomfortable when only owners assess an outcome. The reliability and validity of outcome measures for use by veterinarians for the assessment of lameness,^33^ radiographic changes,^24^, ^34^ and pain^35^ in canine OA are at an early stage and this is a clear area for future research.

While other validated outcome measures were found during the course of our review, they do not appear to be widely adopted.^11^, ^31^, ^36^, ^37^, ^38^, ^39^, ^40^ Few publications included any reference to the need for validation. Fifty publications included a reference to the prior use of an outcome measure, but very few of these outcome measures appeared to have been validated, merely previously used. Barriers to adoption of validated outcome measures by veterinarians are unknown and should be investigated. Such barriers may include a lack of awareness of the value of validation, that validated outcome measures are not measuring the outcomes in which veterinary surgeons are most interested, or that they are not in a format that is easy to use. The COMP initiative^41^ and our review address the first of these potential barriers.

In agreement with the findings of other recent veterinary reviews, methodologies and basic data in many of the publications were poorly reported.^5^, ^42^, ^43^ This is not a problem unique to veterinary orthopedics, as human orthopedic surgeons have commented on the hindrance this presents to the production of evidence‐based recommendations.^44^ The COMP group has published guidance on the use of terminology in orthopedic studies^45^ and on study design and reporting.^41^ It is hoped that these recommendations, in combination with reporting guidelines,^46^, ^47^ will help veterinary researchers and editors improve the quality of future publications. Simple interventions such as enforced, standardized reporting of force plate gait analysis methodology would help enable comparisons to be made between results of studies that use this methodology across all fields of veterinary orthopedics.^1^, ^29^

Our review has several limitations. CAB Abstracts and PubMed were searched, as they have been found to produce the most results when looking for veterinary literature.^48^ More outcome measures could exist in alternative databases or grey literature (literature not formally published in books or journals).^49^ The outcomes assessed and the outcome measures used for these assessments were heterogeneous so there was a need to combine them in some way to allow any form of comparison. A single category therefore included many different outcome measures that were sometimes only loosely similar. The descriptors associated with some individual outcome measures were so diverse that they were difficult to assign to a single category and, as a result, others may have placed them elsewhere. This review included only a very basic assessment of whether the outcome measures had been validated, as a more thorough appraisal was outside its scope, but this would be valuable future work. Due to the length of time taken by the review methodology, additional validated outcome measures have since been produced such as the Canine Orthopedic Index,^30^, ^50^, ^51^ which is designed to complement the validated Canine Brief Pain Inventory (CBPI;^31^ personal communication, DC Brown, February 2014).

The findings of our systematic review suggest that a consensus is needed on the outcomes that should be assessed in canine OA and the validated outcome measures that should be used for those assessments. More work is needed to ascertain which outcomes owners and veterinarians managing dogs with OA find the most useful, how reliably these can be assessed, and whether barriers exist to the adoption of current validated outcome measures. Until a consensus is reached, we recommend inclusion of at least 1 existing, validated outcome measure in each future study. There is an urgent need for the validation of outcome measures for outcomes being frequently assessed by veterinarians. It will not be possible to ascertain the best treatment for canine OA until a systematic review can be performed including publications that measure the same outcomes using the same validated outcome measures.

## Disclosure

The authors declare no conflicts of interest related to this report.
